# Investigation of hippocampal synaptic transmission and plasticity in mice deficient in the actin-binding protein Drebrin

**DOI:** 10.1038/srep42652

**Published:** 2017-02-15

**Authors:** Claudia G. Willmes, Till G. A. Mack, Julia Ledderose, Dietmar Schmitz, Christian Wozny, Britta J. Eickholt

**Affiliations:** 1 Institute of Biochemistry, Charité - Universitätsmedizin Berlin, Charitéplatz 1, 10117 Berlin, Germany; 2 NeuroCure - Cluster of Excellence, Charité - Universitätsmedizin Berlin, Germany; 3 Neuroscience Research Center (NWFZ), Charité Universitätsmedizin, 10117 Berlin, Germany; 4 Strathclyde Institute of Pharmacy and Biomedical Sciences, University of Strathclyde, 161 Cathedral Street, Glasgow, G4 0RE, UK

## Abstract

The dynamic regulation of the actin cytoskeleton plays a key role in controlling the structure and function of synapses. It is vital for activity-dependent modulation of synaptic transmission and long-term changes in synaptic morphology associated with memory consolidation. Several regulators of actin dynamics at the synapse have been identified, of which a salient one is the postsynaptic actin stabilising protein Drebrin (DBN). It has been suggested that DBN modulates neurotransmission and changes in dendritic spine morphology associated with synaptic plasticity. Given that a decrease in DBN levels is correlated with cognitive deficits associated with ageing and dementia, it was hypothesised that DBN protein abundance instructs the integrity and function of synapses. We created a novel DBN deficient mouse line. Analysis of gross brain and neuronal morphology revealed no phenotype in the absence of DBN. Electrophysiological recordings in acute hippocampal slices and primary hippocampal neuronal cultures showed that basal synaptic transmission, and both long-term and homeostatic synaptic plasticity were unchanged, suggesting that loss of DBN is not sufficient in inducing synapse dysfunction. We propose that the overall lack of changes in synaptic function and plasticity in DBN deficient mice may indicate robust compensatory mechanisms that safeguard cytoskeleton dynamics at the synapse.

Dendritic spines are dynamic protrusions^1^ that represent the postsynaptic element of excitatory synapses. They serve as a compartment to convert the presynaptic input via signalling cascades into biochemical and electrical signals^2^. To induce changes and maintain the strength of neuronal connectivity, postsynaptic receptors and actin as the major cytoskeletal component and scaffold proteins need to interact in a precise spatial-temporal pattern^3^,^4^,^5^. Neuronal plasticity is evidenced by activity-dependent accumulation or internalisation of AMPA receptors (AMPA-R) to and from the postsynaptic density (PSD)^6^,^7^ to modify synaptic strength. In this context, actin dynamics appear to be crucially important for receptor trafficking^8^ and structural adaptions^9^,^10^ during synaptic plasticity.

Reorganization of actin filaments is controlled by actin-associated proteins that control filament nucleation, branching, severing, bundling, elongation, and capping^10^ to dynamically alter the morphology of dendritic spines^11^. Highly localised changes in actin dynamics regulate receptor trafficking and ultimately expression of neuronal plasticity^12^. Synaptic stimulation facilitates an intricate pattern of actin remodelling^4^,^13^, which in turn orchestrates the postsynaptic response to neuronal stimuli.

Synaptic plasticity is often accompanied by morphological changes^14^. During long-term potentiation (LTP) and long-term depression (LTD) dendritic spines undergo structural changes such as enlargement and shrinkage, respectively. Notably, although these forms of synaptic plasticity show distinct underlying cellular and molecular mechanisms, both rely on alterations in actin cytoskeleton dynamics. In the initial phase of long term potentiation the amount of actin^15^ and proteins that modify F-actin through severing, branching or capping rapidly increases^4^. This phase is followed by an increase in proteins that are known to stabilize the supra-structure of the actin cytoskeleton by bundling F-actin or linking F-actin to the PSD in the spine^4^. Of note, pathological changes associated with spine morphology and structural plasticity deficits occur in a variety of neurological disorders accompanied by cognitive decline such as seen in Alzheimer’s disease^16^.

Drebrin (Developmentally-regulated brain protein, DBN) is a cytoplasmic actin-filament binding protein highly expressed in neurons and known to stabilize actin filaments^17^. Two isoforms have been identified, DBN E and DBN A, that are transcribed from a single gene through alternative splicing^18^. DBN E primarily promotes neurite-extension during development^19^,^20^ whereas DBN A localizes to dendritic spines^21^,^22^ and is implicated in shaping spine morphology^23^. Overexpression has been shown to cause spine elongation^24^, and, conversely, depletion of DBN using siRNA in neuronal cultures was reported to induce spine shrinkage coinciding with altered electrophysiological properties^25^.

The current picture of the interplay between synaptic activity and DBN dynamics is not completely understood. It has been suggested that LTP inducing stimuli induce a transient exodus and subsequently, re-entry of DBN into dendritic spines^4^,^26^ resulting in a net-increase of DBN in potentiated spines^27^. Likewise blockage of NMDA receptors (NMDA-R) increases the proportion of DBN immuno-positive spines^28^.

Two mouse models of DBN deficiency have been previously published, one being deficient for the isoform conversion of DBN A from DBN E^29^ and a DBN KO mouse generated by excision of DBN exons 4–7^30^. Both models show reduced hippocampal LTP and altered spine morphology in CA1 hippocampal neurons^30^,^31^. Additionally, DBN A deficient mice exhibit impaired context dependent fear conditioning^29^.

We generated novel conditional mouse alleles of the Dbn1 gene and analysed ubiquitous and cell-type specific knockout effects of the actin binding protein DBN. Surprisingly, DBN KO mice develop normally and show no obvious defects in brain development. Deletion of DBN did not alter the basic properties at hippocampal CA1 synapses or different forms of plasticity including LTD, LTP and homeostatic plasticity. Our data argue against a prominent role of DBN in mediating changes in synaptic strength at hippocampal excitatory synapses, at least in the healthy young adult brain.

## Results and Discussion

### Generation of DBN KO mice

**Figure 1:**
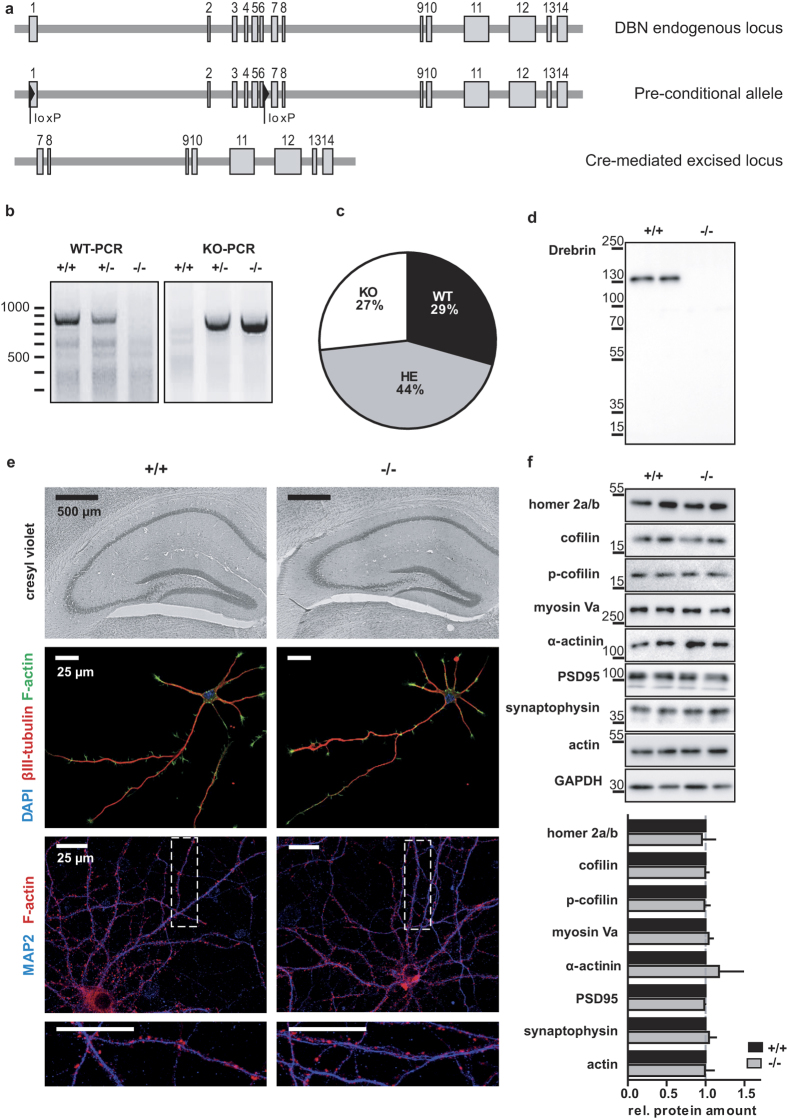
Generation of DBN knockout mice. (**a**) Schematic representations of the mouse Drebrin (DBN) genomic locus, targeted alleles and excision of the coding exons by Cre recombinase. Endogenous DBN contains 14 exons. Protein coding segments are shown as grey rectangles. LoxP sites are indicated by black triangles, The DBN locus after Cre-mediated excision corresponds to the constitutive DBN KO. (**b**) Verification of DBN KO PCR by using two different forward primers, for wild type (WT) in exon 6, for DBN knockout (KO) upstream of exon 1 and one reverse primer in the region between exon 6 and 7, PCR results for WT allele show 844 bp and for KO allele show 650 bp. Heterozygous mice presented bands in both PCR condition. Lines indicate DNA ladder in base pairs (bp). (**c**) Mendelian distribution of DBN deficient progeny (total number of litters analysed: 18; total number of mice: wild type (WT): 32, heterozygous (HET): 46, knockout (KO): 30). (**d**) Representative western blot from WT and KO mouse hippocampus lysates probed with DBN specific peptide antibody recognizing an epitope between Cys651 and Phe662 of the adult isoform. Lines indicate protein ladder in kilodalton. (**e**) Nissl staining showed no gross abnormalities in morphology of hippocampal area. Primary neuronal cultures prepared from DBN KO embryos showed no abnormalities in neurite outgrowth at DIV3 or spine size and number at DIV 21. (**f**) Hippocampus lysates were probed for different pre- and post-synaptic marker proteins as well as actin binding proteins. Western blot band densitometry revealed no altered protein levels for DBN KO samples compared to WT. Data show mean ± s.e.m. of 3 experiments.

The Dbn1 KO mice were established from ES cell clone EPD0211_3_A05, obtained from the supported KOMP Repository (www.komp.org)^32^ generated by the Wellcome Trust Sanger Institute. Following removal of the trapping cassette by crossing with a Flp deleter strain, the resulting pre-conditional allele was validated by PCR and sequencing (data not shown). DBN KO mice were obtained by crossing homozygote pre-conditional mice (Dbn1^loxp/loxp^) with a Cre/loxP-deleter strain, which induced the expected excision of exon 1–6 between loxP sites (Fig. 1a,b; Fig. S1a). As a result of homozygosity for the null allele, full length DBN protein or DBN fragments of any lower molecular weight were not detected (Fig. 1d). This validated the use of our genetically modified mice as a proper tool to study the physiological effects of DBN depletion.

DBN KO mice are viable and fertile, and Mendelian distribution of DBN deficient progeny was as expected (Fig. 1c; number of litters analysed: 18; total number of mice: wild type (WT): 32, heterozygous (HET): 46, knockout (KO): 30). Overall, brain sizes between the two genotypes were comparable and gross brain morphology of WT and DBN KO littermates at P37 revealed no changes in cresyl violet stained brain slices (Fig. 1e upper panel; Fig. S1b). Similarly, the anatomy of the corpus callosum, which is composed of axons that cross the midline and form connections in the contralateral side of the brain, was well preserved in DBN KO brains (Fig. S1b). To further examine neuritogenesis and axon formation, control and DBN KO hippocampal neurons were cultured *in vitro*, which revealed no differences in overall neurite formation, neuronal polarization or axon branching (Fig. 1e second panel). Neurons obtained from DBN KO brains also showed no obvious differences in dendritic spine formation, dendritic spine size or spine number (Fig. 1e lower panel). These results contrast previous studies in neuronal cell culture which linked DBN A as an essential actin regulator to the control of neuritogenesis^19^,^20^ and the establishment of dendritic spine morphology and density^33^,^34^,^35^.

Expression of the postsynaptic density marker PSD-95 or the presynaptic marker protein synaptophysin in hippocampus homogenates of DBN KO mice was equivalent to the expression in WT samples (Fig. 1f). The protein levels of the scaffolding and DBN interacting protein Homer^36^ and the actin binding proteins cofilin^37^, myosin^38^,^39^ and α-actinin^36^,^40^, which have previously been shown to compete with DBN for actin binding sites were not different in hippocampus homogenates generated from WT or Dbn1 KO mice. (PSD-95: WT 1.00 ± 0.03 KO 0.98 ± 0.01, P = 0.66; synaptophysin: WT 1.00 ± 0.12 KO 1.04 ± 0.09, P = 0.79; homer: WT 1.00 ± 0.07, KO 0.95 ± 0.17, P = 0.80; cofilin: WT 1.00 ± 0.03, KO 0.99 ± 0.04, P = 0.93; cofilin-phosphoS3: WT 1.00 ± 0.04, KO 0.98 ± 0.07, P = 0.85; myosin Va: WT 1.00 ± 0.08 KO 1.04 ± 0.06 P = 0.77; α-actinin: WT 1.00 ± 0.27 KO 1.17 ± 0.31, P = 0.69; N = 3; unpaired t-test).

These results indicate that gross brain anatomy and dendritic spine protein composition is not impaired in this newly generated DBN deficient mouse model.

### Basic synaptic transmission is normal in DBN KO mice

**Figure 2:**
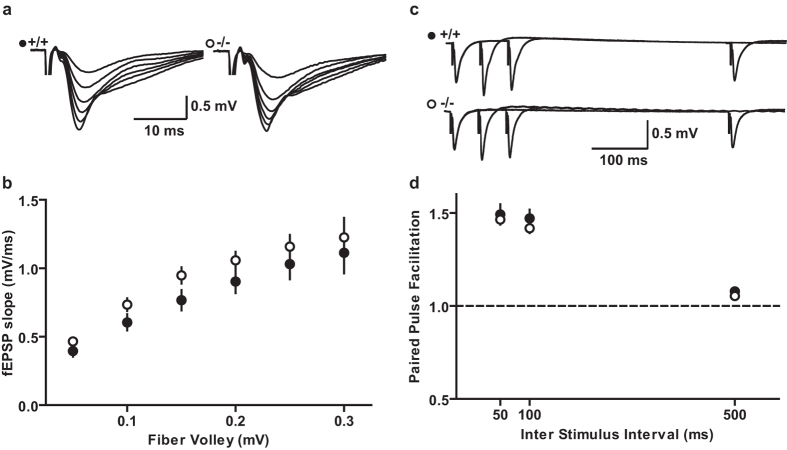
Synaptic responses in area CA1 in WT and DBN KO mice. (**a**) Sample traces of input-output curves in area CA1 of the hippocampus for WT (+/+) and DBN KO (−/−) mice. (**b**) No significant difference was found between WT and DBN KO mice in the fEPSP slopes at various afferent volley amplitudes (two-way ANOVA). (**c**) Sample traces for paired-pulse intervals of 50, 100 and 500 ms. (**d**) Paired-pulse facilitation is unchanged in DBN KO mice compared to WT (two-way ANOVA). Data show mean ± s.e.m.

To investigate the hypothesis that DBN deficiency causes defects in synaptic properties, we first tested whether the loss of DBN leads to alterations in postsynaptic responses. To this end, we performed electrophysiological recordings in acute hippocampal slices in young (P13-P16) and young adult (P34-P39) mice. We stimulated the Schaffer collaterals in area CA1 and recorded postsynaptic field potentials (fEPSPs) in the stratum radiatum of area CA1. Comparing the size of the afferent fibre volley (FV) as a measure for the excitation of presynaptic fibers, with the slope of the fEPSP representing the postsynaptic responsiveness, we found no significant differences between the two groups in their input–output relationship. (Fig. 2a,b; WT N = 3, n = 19; KO N = 3, n = 21; P > 0.05; Fig. S1a,b; WT N = 3, n = 18; KO N = 3, n = 14; P > 0.05, two-way ANOVA, Bonferroni post-hoc test), which is in agreement with previous studies using acute slice preparations of DBN deficient mouse models^30^,^31^.

To study presynaptic effects that may occur as a consequence of DBN deficiencies, we analysed paired-pulse facilitation (PPF). Using stimulation intervals at 50, 100 and 500 ms, we found that PPF is not significantly changed in DBN KO mice (Fig. 2c,d; WT, N = 3, n = 13; KO, N = 3, n = 17; P > 0.05; Fig. S1c,d; WT N = 3, n = 13, KO N = 3, n = 17; P > 0.05, two-way ANOVA, Bonferroni post-hoc test). These results suggest that the synaptic connections between Schaffer collaterals and CA1 pyramidal cells are functionally normal in DBN KO mice.

### Single cell DBN KO does not have an impact on AMPA-R mEPSCs

**Figure 3:**
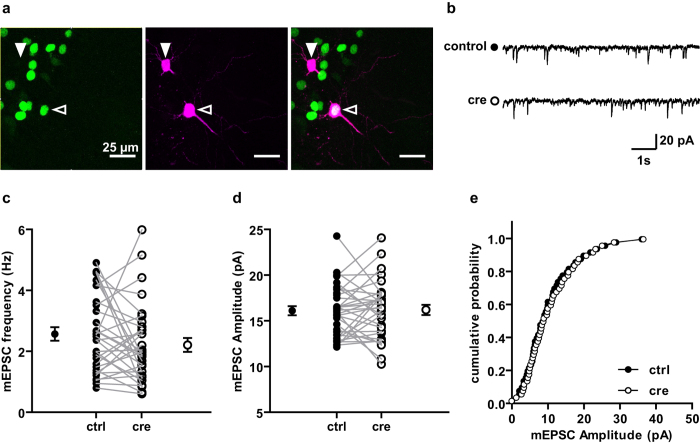
Single cell DBN KO does not have an impact on AMPA R mEPSCs. (**a**) Representative image of CA1 pyramidal neurons in DBN^loxp/loxp^ slices infected with Cre-eGFP-NLS AAV (green). Biocytin labelling (magenta) of recorded neurons distinguished infected (open arrow) from non infected neurons (closed arrow). (**b**) mEPSCs in slices were recorded in the presence of TTX (1 μM), gabazine (1 μM), D-APV (50 μM) and cyclothiazide (100 μM). (**c**) mEPSCs showed no change in frequency (paired t-test). Mean values of paired cells are plotted with connected lines, outer circles represent mean ± s.e.m. (**d,e**) mEPSC amplitudes calculated from 50 random events of a 1 min recording per cell in each experimental condition were not significantly different in the mean nor the cumulative distribution (P > 0.05, KS normality test, Wilcoxon Signed Rank Test).

To differentiate between network and single-cell phenotype, the *dbn1* gene was deleted in a subset of hippocampal pyramidal cells that normally express DBN protein. This was achieved by viral mediated co-expression of Cre-recombinase with a GFP-tagged nuclear localisation signal in DBN^loxp/loxp^ mice. Injection led to sparse labelling of CA1 hippocampal neurons (Fig. 3a), whereas the Schaffer collaterals forming synapses onto DBN deficient CA1 pyramidal cell derived from WT neurons. Subsequent recordings from GFP^+^ (DBN KO genotype) and GFP^−^ (WT genotype) CA1 pyramidal neurons in acute hippocampal slices were performed at P21 ± 3 days. Miniature excitatory postsynaptic currents (mEPSCs) were recorded in the presence of the sodium channel blocker tetrodotoxin (TTX), the AMPA-R desensitization inhibitor cyclothiazide, the GABA_A_ receptor antagonist gabazine (SR 95531), blocking any contribution of fast inhibitory synaptic currents, and APV, a selective NMDA-R antagonist. CA1 pyramidal cell identity was verified post-hoc by biocytin staining of the recorded cell (Fig. 3a,b). Infected neurons showed spontaneous activity demonstrating that neurons were vital and fully integrated in the neuronal network. Sequential recordings from a GFP^+^ and a GFP^−^ CA1 pyramidal cell (or vice versa) showed neither significant differences in mEPSC frequency (Fig. 3c; GFP^−^ 2.6. ± 0.2 Hz; GFP^+^ 2.2 ± 0.2 Hz; N = 15, n = 32; P = 0.17; paired t-test) nor in the mean amplitude (Fig. 3d; GFP^−^ 16.1 ± 0.5 pA; GFP^+^ 16.2 ± 0.6 pA; N = 15, n = 32; P = 0.85; paired t-test). The cumulative probability plots of the mEPSC amplitudes were not significantly shifted (Fig. 3e; P > 0.05, KS normality test, Wilcoxon Signed Rank Test), indicating that DBN deficiency does not impact AMPAR-mediated transmission. In combination with the fact that frequency remained unchanged, we conclude that neurotransmitter release is not altered and thus presynaptic mechanisms are not affected by loss of DBN.

### Long-term plasticity is not impaired in DBN KO mice

Two recent studies reported that DBN and DBN A deficient mice exhibit impaired LTP^30^,^31^ and hippocampus-dependent learning tasks^30^,^32^.

Induction of LTP and LTD requires NMDA-R activation^41^,^42^,^43^ whereas continued expression depends on recruitment and internalisation of AMPA-R^43^. As DBN was reported to facilitate synaptic targeting of NMDA-R^44^,^45^, we tested if loss of DBN might cause impairments in various forms of synaptic plasticity.

**Figure 4:**
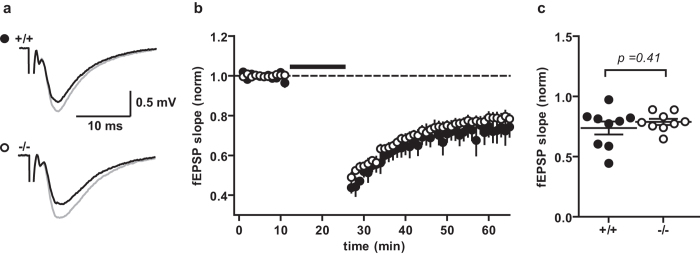
Long term depression is not impaired in DBN KO mice. (**a**) Example traces of WT (+/+) and KO (−/−) mice averaged from baseline (grey) and the last 5 minutes after LTD induction (black). (**b**) Average time course of the slope of field excitatory potentials (fEPSPs) normalized to the baseline. Line: LTD induction (900 pulses at 1 Hz for 15 minutes). Average fEPSP slopes of the last 5 minutes (**c**) showed no difference between WT and KO (unpaired t-test). Data show mean ± s.e.m.

To study effects on long-term synaptic plasticity in WT and DBN KO mice, we recorded a 10–15 min baseline of stable fEPSPs in area CA1 before applying plasticity induction protocols. To induce LTD, the Schaffer collaterals were stimulated with 900 pulses at 1 Hz for 15 minutes. No significant differences between WT and DBN KO littermates were found in the fEPSP slope 30–35 min after induction of LTD (Fig. 4a,c; WT 74% ± 5%, N = 3 n = 9; KO 78% ± 2%, N = 3 n = 9; P = 0.41, unpaired t-test).

**Figure 5:**
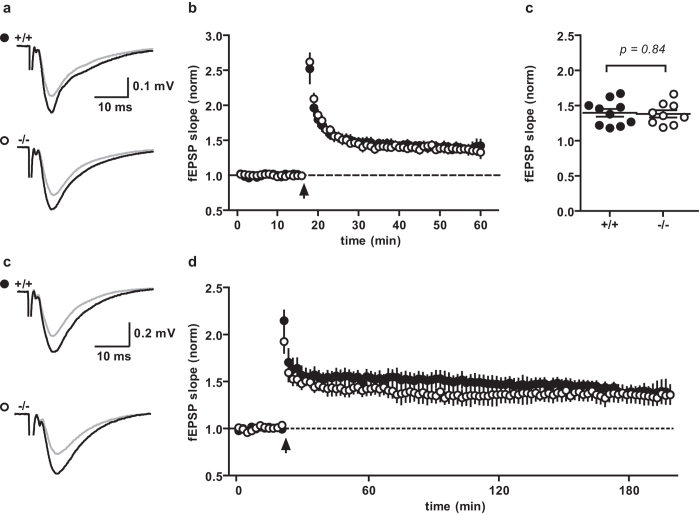
Long term potentiation is not impaired in DBN KO mice. (**a**) Example traces from WT (+/+) and KO (−/−) mice pre (grey) and 45 min post (black) LTP induction performed in ACSF supplemented with glycine (10 μM) and D-serine (20 μM) at room temperature and a superfusion of 5 ml min-1. (**b**) Average time plot of the slope of field excitatory potentials (fEPSPs) normalized to the baseline with a binning of 1 min. Arrow: LTP induction. (**c**) Average fEPSP slopes of the last 5 minutes showed no difference between WT and KO (unpaired t-test). Data show mean ± s.e.m. (**d**) Example traces from WT (+/+) and KO (−/−) mice pre (grey) and 180 min post (black) LTP induction recorded at 29 ± 0.5 °C with superfusion of 2 ml min^−1^. (**e**) Average time course normalized to the baseline with a binning of 2 min.

Next, we tested if loss of DBN affects the continuous process of induction and expression of LTP. Maintenance of LTP is highly dependent on external parameters such as oxygenation and temperature^46^,^47^. Therefore we tested different conditions that varied the temperature (potentially influencing actin dynamics) and the flow rate of recording solution (potentially affecting oxygenation and/or the washout of secreted factors^48^), or the composition of the recording solution (addition of glycine and D-serine as co-agonist of NMDA receptors). In the first approach we recorded at room temperature, a perfusion rate of 5 ml min^−1^ to avoid anoxia and provided glycine (10 μM) and D-serine (20 μM) in the medium. No significant differences in potentiation were detectable between WT and DBN KO slices in the fEPSP slope 40–45 minutes after induction of LTP (Fig. 5a,c; WT 139% ± 6%, N = 6 n = 10; KO 138% ± 5%, N = 5 n = 9; P = 0.84, unpaired t-test). In the second approach, we applied a protocol that had previously been reliably used to investigate long-lasting forms of LTP (l-LTP; >2 h) consisting of elevated temperature (29 ± 0.5 °C) and a perfusion rate of 2 ml min^−1^. Again, the level of potentiation did not differ between slices from WT and KO mice 40–45 min post LTP induction (Fig. 5d; WT 161% ± 13%, N = 4, n = 5; KO 148% ± 10%, N = 3, n = 4; P = 0.48, unpaired t-test). Recordings from DBN KO mice also showed stable l-LTP (WT 137% ± 4%, N = 5 n = 5; KO 134% ± 8%, N = 5 n = 5; P = 0.72, unpaired t-test) indicating no additional role of DBN in extending synaptic enhancement from a short-lasting to a long-lasting form.

Taken together our results show that neither LTD nor LTP are altered in DBN KO mice. DBN was shown to be involved in the spatiotemporal reorganization during LTP as a solidifier^4^, yet our data show that the loss of DBN is dispensable for the long-lasting maintenance of LTP at the Schaffer-collateral-CA1 synapse.

### Homeostatic plasticity is not impaired in DBN deficient primary neuronal cultures

**Figure 6:**
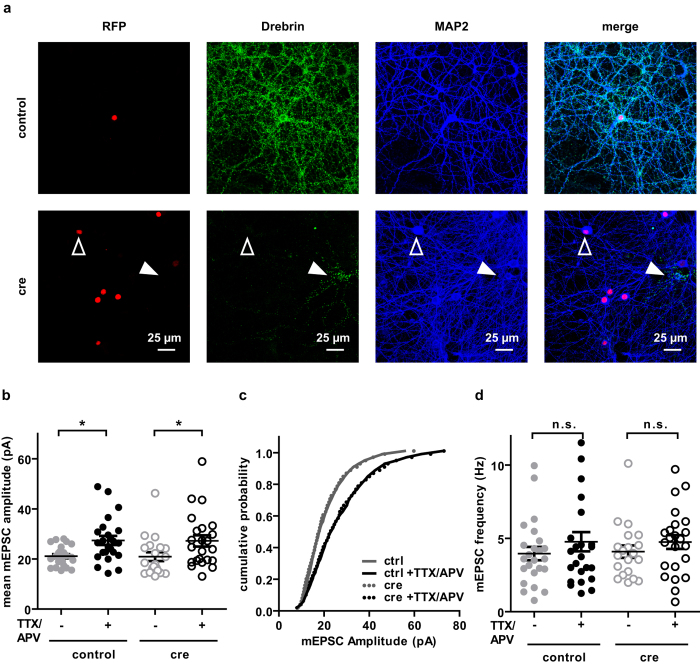
Deletion of DBN does not affect the homeostatic synaptic scaling. Cultured hippocampal neurons from DBNfl/fl mice were infected with Cre-RFP-NLS or RFP-NLS lentivirus at DIV 7. (**a**) Exemplary images of neurons infected with RFPnls (control, first row) or Cre-RFPnls (cre, second row) seven days after viral infection, stained with anti-DBN and anti-MAP2 antibody. Open arrow points at RFP+ neuron, in which DBN (green) has been deleted, filled arrow points at uninfected neuron stained positive for DBN. Cultures were silenced for 24 ± 4 h with TTX (1 μM) and APV (100 μM). mEPSCs were recorded in the presence of TTX (1 μM) and picrotoxin (100 μM). (**b**) The mean mEPSC amplitude calculated from 50 random events of 1 minute for each recording was significantly increased after chronic silencing of synaptic activity in control (P < 0.05, one-way ANOVA, Bonferroni post-hoc test) and Cre-infected neurons (P < 0.05, one-way ANOVA, Bonferroni post-hoc test). Data show mean ± s.e.m. Analysis of the cumulative probability (**c**) did not reveal significant differences (P > 0.05, KS normality test, Wilcoxon Signed Rank Test). (**d**) mEPSC frequency remained unchanged for all conditions (P > 0.05, one-way ANOVA, Bonferroni post-hoc test). Data show mean ± s.e.m.

Finally, we tested DBN deficient neurons for another form of synaptic plasticity: Homeostatic synaptic plasticity affects synaptic networks globally within a desired range in a compensatory manner and guards the network from hyper-excitation or silencing^49^. The process involves postsynaptic trafficking of glutamate receptors^49^. Since DBN is able to remodel the actin cytoskeleton – the scaffold for traffic of vesicular cargo – we hypothesised that DBN deficiency could interfere with transport of AMPA-R-loaded vesicles during homeostatic scaling in response to chronic synaptic silencing. Furthermore, DBN was reported to facilitate activity dependent NMDA-R insertion^44^ whereas depletion of DBN A diminishes the homeostatic synaptic increase in NR2A upon NMDA-R inhibition^50^. TTX, a potent Na^+^ channel blocker and D-APV, a selective NMDA-R antagonist were added to neuronal cultures for 24 ± 4 hours (h) to block action potentials and chronically silence network activity. Under these conditions, induced homeostatic synaptic plasticity is evidenced by an increase in mEPSC amplitudes^49^,^51^. To isolate AMPA-R-mediated mEPSCs from sister DBN^loxp/loxp^ cultures infected with Cre or control virus (Fig. 6a), we included TTX and picrotoxin in the recording solution to block action potentials and GABAergic currents, respectively.

Untreated DBN positive neurons had an average amplitude of 21.1 ± 0.8 pA (N = 4, n = 24), which was significantly increased after chronic silencing of synaptic activity (Fig. 6b left panel; 27.4 ± 1.8 pA, N = 4, n = 24; P < 0.05 compared to silenced DBN positive neurons; one-way ANOVA, Bonferroni post-hoc test). However, the silencing effect was not significantly different in Cre-infected, DBN deficient neurons (Fig. 6b right panel; basal 20.9 ± 1.7 pA, N = 4 n = 20; silenced 27.2 ± 2.2 pA, N = 4, n = 23; one-way ANOVA, Bonferroni post-hoc test). Analysis of the cumulative probability of amplitudes did not reveal any significant changes either (Fig. 6c; P > 0.05, KS normality test, Wilcoxon Signed Rank Test). The frequency of mEPSCs remained unchanged for all conditions (Fig. 6d; basal, DBN^+^ 4.1 ± 0.5 Hz; silenced, DBN^+^ 4.1 ± 0.5 Hz; basal DBN^−^ 4.1 ± 0.4 Hz; silenced DBN^−^ 4.8 ± 0.5 Hz; one-way ANOVA, Tuckey’s Multiple Comparison Test).

These results demonstrate that chronic synaptic silencing did not affect DBN KO cultures differently than WT cultures. Depletion of DBN does not perturb the physiological expression of increased synaptic strength as a result of chronic TTX and APV induced homeostatic synaptic plasticity in primary neuronal cultures.

## Conclusion

In summary, in none of the five sets of electrophysiological experiments presented in this study we observed changes between WT and KO animals in glutamatergic transmission as a result of altered synaptic drive. Our results appear to contradict previous *in vitro*^25^, *ex vivo*^30^,^31^ and *in vivo*^30^,^32^,^52^ experiments, which might be due to different methodological approaches. However, we effectively controlled for the validity of our KO model and carefully exercised different protocols to confirm our findings.

The finding of no discernible phenotype might be due to two possibilities^53^: (i) the intrinsic feature of genetic robustness in actin binding proteins enables genetic buffering by alternative pathways or functional complementation of other genes, or (ii) the abnormal phenotype will only become evident under specific conditions, for example ageing, disease or stress.

The lack of phenotypic changes in glutamatergic synaptic hippocampal synapses of DBN KO mice is possibly caused by compensatory mechanisms as functional redundancy and overlapping functions have been reported for several actin binding proteins^54^.

It is worth speculating that under certain conditions, the loss of DBN cannot be compensated, unmasking a specific DBN KO phenotype. In this regard, cellular stress^55^ or aging might render the dendritic spine vulnerable to the loss of the actin binding protein DBN. However, at this point, our results support the idea that DBN deficiency alone is not sufficient in causing synaptic dysfunction.

## Methods

All animals used were handled in accordance with the relevant guidelines and regulations. Protocols were approved by the ‘Landesamt für Gesundheit und Soziales’ (LaGeSo; Regional Office for Health and Social Affairs) in Berlin and animals reported under the permit number T0347/11 and G0189/14.

### Generation of DBN KO Mice

Heterozygous mice harbouring a promoter-driven Knockout First allele (Dbn1tm1a(KOMP)Wtsi) were obtained from the KOMP Repository. The trapping cassette creating a constitutive null mutation was removed by crossing with a Flp deleter strain and the resulting pre-conditional allele was validated by PCR and sequencing (data not shown) and subsequently bred to homozygosity. Primary neuronal cultures of homozygote pre-conditional embryos were infected with Cre-recombinase and loss of DBN protein was apparent (data not shown). Subsequently, homozygote pre-conditional mice were crossed with Cre/loxP-deleter mice (B6.C-Tg(CMV-cre)1Cgn/J, Jackson Laboratories). Conversion of pre-conditional alleles into null-alleles was monitored by PCR-based genotyping. Subsequently, wild-type and knockout littermates from heterozygous crossings were used for experiments.

Genotyping Primers (5′−3′):

F4: CGCCGGAACCGAAGTTCCTATT (KO-PCR forward primer upstream of exon1)

F3: GAGGAGGTTAAAGGAGCAGTCTATCTTT (WT-PCR forward primer end of exon 6)

RS1: AGGAATACTCAAGTTCCTGTCGGACC (reverse primer between exon 6 and 7)

### Generation of rAAV and Stereotaxic Injections

To induce sparse DBN KO in hippocampal neurons, stereotaxic rAAV injections into neonate DBN^loxp/loxp^ mice at P0 were performed as described previously^56^ with the following coordinates relative to lambda: Anteriorposterior: −2.0mm mediolateral: −0.7 mm dorsoventral: +1.6 mm. 1 μl virus per hemisphere was injected at 30 nl s^−1^ through a 33 G needle via a Nanofil syringe (World Precision Instruments, Inc., Sarasota, FL, USA). After recovery from anaesthesia, the pups were returned into their original litter and kept until experiments were performed starting from P21. The CAG-eGFP-CreNLS recombinant AAV gene transfer vector was generated by the viral core facility Charité – Universitätsmedizin, Berlin, Germany.

### Cell Culture

Primary hippocampal neuronal cultures were prepared as described previously^57^ from E16.5 DBN fl/fl embryos. For electrophysiology, cells were plated in Neurobasal-A supplemented with 1.6% DMEM High Glucose, 5.4% FCS, 2% B27 and 0.2% penicillin/streptomycin. Neurons were plated at a density of ~120,000 cells per 3.69 cm^2^ and kept at 95% O_2_, 5% CO_2_ and 37 °C. After 12 h 80% of serum-media was replaced with Neurobasal-A supplemented with 2% B27, 0.2% penicillin/ streptomycin and 1% Glutamax.

For viral transduction, a lentiviral vector expressing RFP-Cre or RFP with a nuclear localisation (nls) site under the synapsin-1 promotor was used. Lentiviruses were produced as described previously^57^. Cultures were infected with Cre-RFPnls or RFPnls lentivirus at day *in vitro* (DIV) 7. To induce homeostatic synaptic scaling the medium was supplemented with 1 μM tetrodotoxin (TTX) and 100 μM D-APV for 24 ± 4 h as described previously^58^. Recordings were performed at DIV14 – DIV17.

### Slice Preparation

Acute hippocampal slices were prepared as described previously^59^ from 2–6 weeks old littermates of both genders in protective cutting solution. For field recordings, slices were incubated for 30 minutes (min) in sucrose-based ACSF (sACSF) containing (in mM) 87 NaCl, 1.25 NaH_2_PO_4_, 2.5 KCl, 26 NaHCO_3_, 7 MgCl_2_, 0.5 CaCl_2_, 75 saccharose and 25 glucose at 35 °C and subsequently stored in ACSF containing (in mM) 119 NaCl, 26 NaHCO_3_, 10 glucose, 2.5 KCl, 1 NaH_2_PO_4_, 2.5 CaCl_2_ and 1.3 MgCl_2_ (300 mOsm) at room temperature and experiments were commenced after 30 min at the earliest. For whole cell recordings, slices were stored in an interface chamber at 33 ± 1 °C and perfused with ACSF in 1 ml min^−1^. Slices were allowed to recover for at least 1 h after preparation. All ACSF solutions were constantly oxygenated with carbogen (95% O_2_, 5% CO_2_).

### Electrophysiology

Recordings were performed with a MultiClamp 700B (Axon Instruments, Union City, CA, USA) amplifier, signals were filtered at 2 kHz and digitized (National Instruments, BNC-2090) at 5 kHz, recorded and analysed with custom-made software in IGOR Pro (WaveMetrics Inc., OR, USA). Slice recordings were performed submerged in ACSF equilibrated with carbogen (95% O_2_, 5% CO_2_) at room temperature perfused at 5 ml min^−1^ unless stated otherwise. fEPSPs were recorded with low-resistance patch-clamp electrodes filled with ACSF in the CA1 stratum radiatum. For extracellular fibre stimulation, pipettes were placed in the CA1stratum radiatum at the same height as the recording pipette 200 μm apart.

Schaffer collaterals were stimulated with 100 μs pulses at 0.05 Hz. fEPSP magnitude was determined by analysing 20–80% of the amplitude slope. Recordings were only analysed if the fibre volley remained constant throughout the recording. Paired-pulse facilitation was investigated by analysing the ratio of the second to the first fEPSP. For plasticity induction, the simulation intensity was set to elicit 40–50% of the maximum amplitude. Following a baseline of at least 10 min, LTD was induced with 900 pulses at 1 Hz for 15 min, LTP was induced by four tetany of 100 Hz for 1 second in 20 s intervals. LTD was studied in P13-P16 mice to reliably induce NMDA-R dependent synaptic depression^3^, LTP was induced in P34-39 mice.

Whole cell patch-clamp recordings were performed at 31–32 °C. Patch-clamp electrodes (resistance 2–5 MΩ) were filled with internal solution containing (in mM) 130 KMSO_3_, 10 KCl, 4 NaCl, 10 HEPES, 4 Mg-ATP, 0.5 Na_3_GTP, 5 phosphocreatine, 0.25% biocytin; pH adjusted to 7.3 with KOH. Access resistance (6–20 MΩ) was continuously monitored throughout the experiment. Recordings were discarded when the series resistance changed more than 20%. No R_s_ compensation was used. mEPSCs were isolated at −60 mV in the presence of 1 μM TTX, 1 μM gabazine, 50 μM D-APV and 100 μM cyclothiazide. Only slices were analysed, in which mEPSCs were recorded from both infected and uninfected neurons. Putative interneurons were excluded from analysis based on morphology after visualization of biocytin.

For recording in cultures, the chamber was perfused with base + solution containing (in mM) 140 NaCl, 2.4 KCl, 10 HEPES, 2 CaCl_2_, 4 MgCl_2_, 10 Glucose (pH adjusted to 7.3 with NaOH, 300 mOsm ± 10 mOsm.) with addition of 1 μM TTX and 100 μM picrotoxin at 31–32 °C and 3 ml min^−1^. Patch-clamp electrodes were filled with internal solution containing (in mM) 117.5 Cs-gluconate, 2.5 CsCl, 8 NaCl, 10 HEPES, 10.0 TEA, 0.2 EGTA, 4 Mg-ATP, 0.3 Na_3_GTP and QX-314 2; pH adjusted to 7.2 with CsOH.

Cells were identified using infrared differential contrast microscopy (BX51WI, Olympus). Fluorescently labelled cells were identified using fluorescence microscopy (XM10, Olympus) and adequate fluorescence filters visualizing GFP-tagged or RFP-tagged neurons.

Data were analysed with Igor plug-in NeuroMatic (www.neuromatic.thinkrandom.com). For mEPSC analysis, signals were detected automatically and additionally sorted manually by visual inspection to exclude false positive events.

### Drugs and Chemical Compounds

D-APV, D-serine, gabazine, glycine, PTX and TTX were added from aqueous stock solutions; cyclothiazide was administered from a stock solution in DMSO (final concentration of DMSO < 0.1%). Drugs were purchased from Tocris/BioTrends GmbH or Biozol GmbH (Enching, Germany) and Sigma-Aldrich. ACSF compounds from Roth (Karlsruhe, Germany). Cell culture reagents were purchased from Life technologies (Grand Island, NY, USA).

### Histochemistry and Microscopic Quantification

After recording, slices were fixed in 4% paraformaldehyde (PFA) in 0.1 M phosphate buffer (PBS) over night. Biocytin-filled cells were visualized by streptavidin conjugated to Alexa 647 (diluted 1:500 in PBS, 1% Triton) and slices were mounted in Fluoroshield (Sigma-Aldrich, Munich, Germany).

Neuronal cultures were fixed in 4% PFA in PBS for 20 min, washed with PBS and blocked in blocking buffer (0.5% Triton X-100, 2% bovine serum albumin, 1% goat serum in PBS) for 1 h, incubated with primary antibodies (Table S1) and sequentially with secondary antibodies donkey anti mouse Alexa 488 (Jackson ImmunoResearch Inc., PA, USA), donkey anti guinea pig Cy5 (Jackson ImmunoResearch Inc.), goat anti rabbit Alexa 488 (Invitrogen, Germany) and/or actistain (Cytoskeleton Inc., Denver, CO, USA). Afterwards sections were washed and mounted with Mowiol. Images were obtained on a Leica TCS SP5 confocal microscope (Leica, Wetzlar, Germany).

For analysis of brain morphology, P33-P37 mice were anaesthetized with isoflurane, transcardially perfused with 0.1% PBS and subsequently with 4% PFA in 0.1 M phosphate buffer (PB, pH 7.2). Brains were post-fixed in 4% PFA overnight at 4 °C, soaked in 30% sucrose overnight, sectioned in 60 μm slices with a microtome (Thermo Scientific, Microm HM340 E) and processed with cresyl-violet or haematoxylin-eosin.

### Brain lysates and Western blotting

Hippocampi of WT and DBN KO mice (4 to 5 weeks old) were homogenized in RIPA buffer (50 mM Tris-HCl pH 7.4, 1% NP-40, 0,5% Na-deoxycholate, 0,1% SDS, 150 mM NaCl) with freshly added protease and phosphatase inhibitor cocktails (Sigma-Aldrich).

Determination of protein concentration, SDS-PAGE and Western blots were performed as described previously^60^. Primary antibodies are listed in Table S1. Western blot results were quantified with Fiji^61^.

### Data Analysis and Statistics

Statistical analysis was performed using GraphPad Prism 5 (Graph Pad Software, La Jolla, USA), significance level was set to P < 0.05. Data are expressed as mean ± s.e.m. N describes the number of mice or independent cultures; n number of slices or cells. For cumulative probability plots, random 50 events of 1 minute recording per cell were sorted in ascending order and means were calculated for each row. Sample traces are averages of 5–10 consecutive responses, stimulus artefacts were removed.

## Additional Information

**How to cite this article**: Willmes, C. G. *et al*. Investigation of hippocampal synaptic transmission and plasticity in mice deficient in the actin-binding protein Drebrin. *Sci. Rep.* **7**, 42652; doi: 10.1038/srep42652 (2017).

**Publisher's note:** Springer Nature remains neutral with regard to jurisdictional claims in published maps and institutional affiliations.

## Supplementary Material

Supplementary Figure S1 , S2, Table S1
