# Supplementary material for: Investigation of hippocampal synaptic transmission and plasticity in mice deficient in the actin-binding protein Drebrin
Source: Sci Rep. 2017 Feb 15;7:42652. doi: 10.1038/srep42652 (PMC5309812; doi:10.1038/srep42652)
Supplement: Supplementary Figure S1 , S2, Table S1 [file srep42652-s1.pdf]

# Investigation of hippocampal synaptic transmission and plasticity in mice deficient in the actin-binding protein Drebrin

Claudia G. Willmes[1, 2], Till G. A. Mack[1], Julia Ledderose[1], Dietmar Schmitz[2, 3], Christian Wozny[4, a] and Britta J. Eickholt[1, 2, a]

## Supplementary Information

**Table S1. Primary antibodies for immunohistochemistry and immunoblotting**

| Antigen | Species | Dilution | Source |
|---|---|---|---|
| actin | m | 1:3000 (WB) | Genetex – GTX82559 |
| α-actinin | m | 1:800 (WB) | Sigma – A5044 |
| Cofilin | rb | 1:500 (WB) | Cell Signaling – #3312 |
| Cofilin Phospho S3 | rb | 1:500 (WB) | Cell Signaling – #3311 |
| DBN | m | 1:1000 (WB)<br>1:200 (IHC) | Genetex - GTX12350 |
| DBN | rb | 1:1000 (WB) | homemade (see below) |
| GAPDH | m | 1:4000 (WB) | Calbiochem – CB1001 |
| Homer | rb | 1:1000 (WB) | kind gift of Teiichi Furuichi (Laboratory for Molecular Neurogenesis, RIKEN Brain Science Institute, Japan ) |
| MAP2 | gp | 1:200 (IHC) | Synaptic systems – 188004 |
| Myosin Va | rb | 1:1000 (WB) | Cell Signaling – 3402 |
| PSD95 | m | 1:1000 (WB) | Thermo Scientific – MA 1-045 |
| Synaptophysin | m | 1:2000 (WB) | Synaptic systems – 101011 |
| Neuronal Class III β-Tubulin | rb | 1:500 (IHC) | Covance - PRB-435P |

**Pan-DBN Peptide Antibodies**

Pan-DBN Peptide Antibodies were generated from rabbits immunized with a peptide comprising the conserved phospho-site S647 of DBN A (H2N-CGYFSQS(PO3H2)QEEEF-CONH2). Antibodies were purified as described previously [57] and antibodies that bound the non-phosphorylated control peptide (H2N-CGYFSQSQEEEF-CONH2) were referred to as pan-DBN.

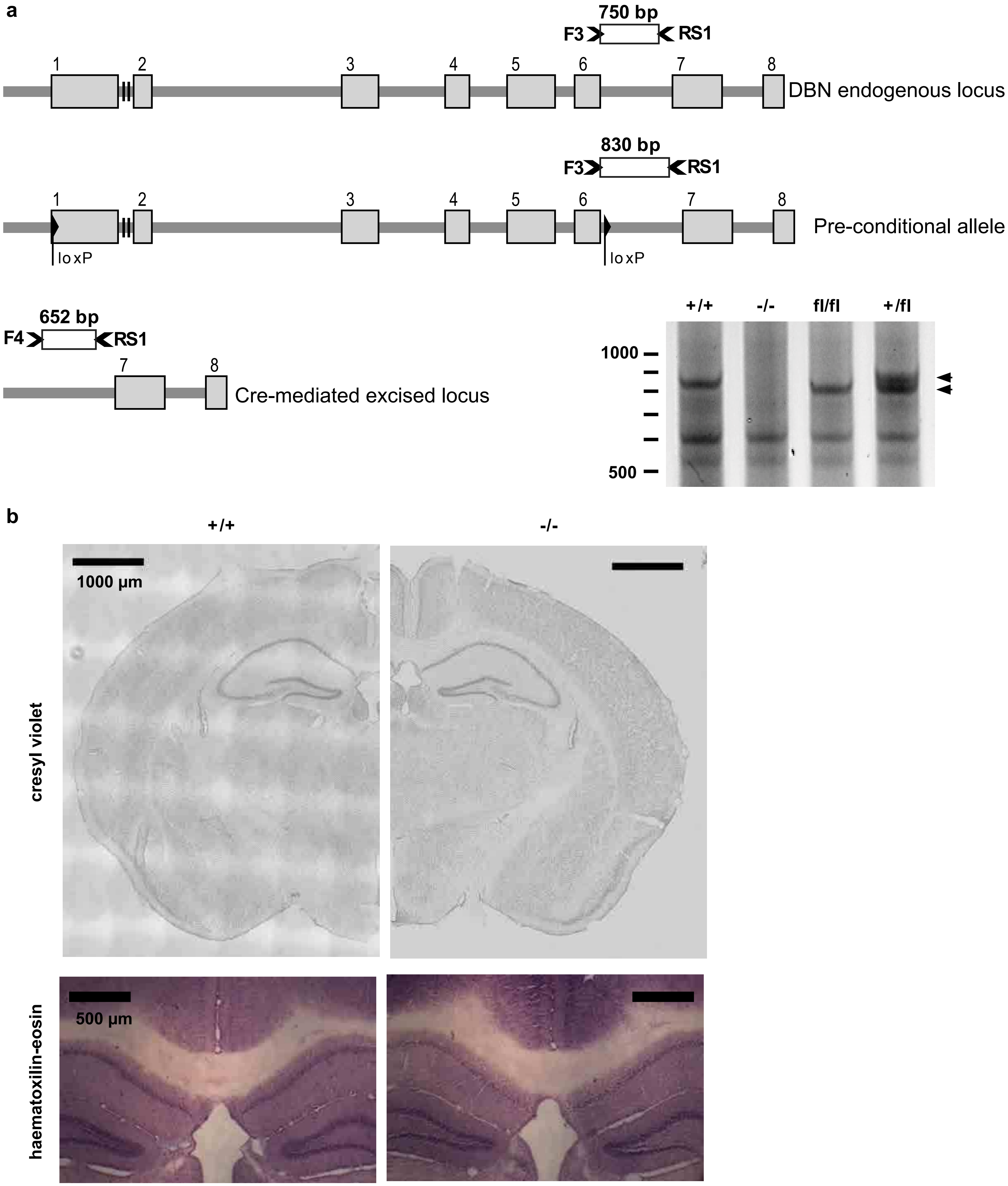
a
750 bp
F3
RS1
1
2
3
4
5
6
7
8
DBN endogenous locus
830 bp
F3
RS1
Pre-conditional allele
loxP
loxP
652 bp
F4
RS1
Cre-mediated excised locus
+/+
-/-
fl/fl
+/fl
1000
500
b
+/+
-/-
1000 µm
cresyl violet
500 µm
haematoxilin-eosin

**Fig. S1. Generation of DBN knockout mice.**

(a) Magnification of DBN exon 1 – 8. Genotyping primers are indicated as black arrows, calculated PCR products are indicated as rectangles, bp: base pairs. Pre-conditional alleles were detected by PCR products with forward primer in exon 6. In contrast, null alleles produced no PCR product under these conditions, but were recognized using a forward primer upstream of exon 1. Agarose gels shows WT PCR with Primer pair F3-RS1 for WT (+/+), DBN KO (-/-), double floxed (fl/fl) and heterozygous WT, floxed (+/fl) mice. The PCR product of the floxed gene is shorter than the estimated size. PCR results for WT allele show 845 bp and for floxed allele show 830 bp. Heterozygous mice presented bands in both PCR condition. Lines indicate DNA ladder in base pairs.

Identity of the PCR products was identified by sequencing (data not shown). (b) Nissl staining showed no gross abnormalities in brain morphology, haematoxilin and eosin staining revealed no differences in the morphology of corpus callosum.

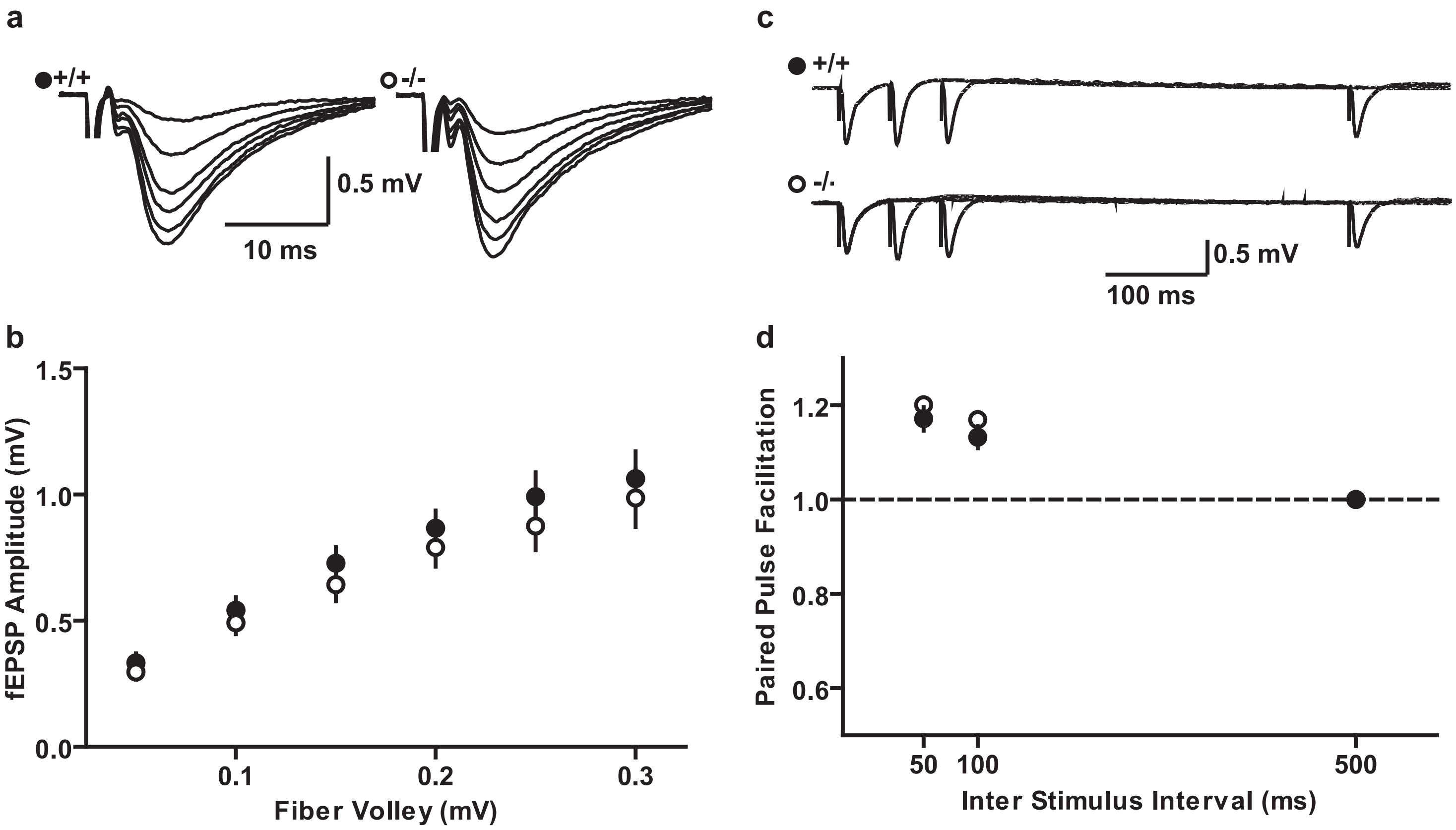

a
+/+
-/-
0.5 mV
10 ms
c
+/+
-/-
0.5 mV
100 ms
b
fEPSP Amplitude (mV)
1.5
1.0
0.5
0.0
0.1
0.2
0.3
Fiber Volley (mV)
d
Paired Pulse Facilitation
1.2
1.0
0.8
0.6
50
100
500
Inter Stimulus Interval (ms)

**Fig. S2. Synaptic responses in area CA1 in young WT and DBN KO mice.**

(a) Sample traces of input-output curves in area CA1 of the hippocampus for WT (+/+) and DBN KO (-/-) mice. (b) No significant difference was found between WT and DBN KO mice in the fEPSP slopes at various afferent volley amplitudes (WT, N=3 n=18; KO, N=3 n=14; $P > 0.05$, two-way ANOVA, Bonferroni post-hoc test). Data show mean ± s.e.m. (C) Sample traces for Paired-pulse intervals of 50, 100 and 500 ms. (D) Paired-pulse facilitation is unchanged in DBN KO mice compared to WT (WT, N=3 n=18; KO, N=3 n=14; $P > 0.05$, two-way ANOVA, Bonferroni post-hoc test).
